# Whole Body Vibration Exercises and the Improvement of the Flexibility in Patient with Metabolic Syndrome

**DOI:** 10.1155/2014/628518

**Published:** 2014-09-03

**Authors:** Danúbia da Cunha Sá-Caputo, Pedro Ronikeili-Costa, Rafaelle Pacheco Carvalho-Lima, Luciana Camargo Bernardo, Milena Oliveira Bravo-Monteiro, Rebeca Costa, Janaina de Moraes-Silva, Dulciane Nunes Paiva, Christiano Bittencourt Machado, Paula Mantilla-Giehl, Adriano Arnobio, Pedro Jesus Marin, Mario Bernardo-Filho

**Affiliations:** ^1^Mestrado Profissional em Saúde, Medicina Laboratorial e Tecnologia Forense, Universidade do Estado do Rio de Janeiro, 20950003 Rio de Janeiro, RJ, Brazil; ^2^Departamento de Biofísica e Biometria, Instituto de Biologia Roberto Alcantara Gomes, Universidade do Estado do Rio de Janeiro, Avenida 28 de Setembro, 87 fundos, 4° Andar, Vila Isabel, 20551030 Rio de Janeiro, RJ, Brazil; ^3^Programa de Pós-Graduação em Ciências Médicas, Universidade do Estado do Rio de Janeiro, 20550170 Rio de Janeiro, RJ, Brazil; ^4^Departamento de Fisioterapia, Faculdade Maurício de Nassau/Aliança, 64049240 Teresina, PI, Brazil; ^5^Programa de Pós-Graduação em Promoção da Saúde, Universidade Santa Cruz do Sul, 96815900 Santa Cruz do Sul, RS, Brazil; ^6^Laboratório de Ultrassom Biomédico, Universidade Estácio de Sá, 20261063 Rio de Janeiro, RJ, Brazil; ^7^Laboratorio de Fisiología, Universidad Europea Miguel de Cervantes, Valladolid, 47012 Castile and León, Spain

## Abstract

Vibrations produced in oscillating/vibratory platform generate whole body vibration (WBV) exercises, which are important in sports, as well as in treating diseases, promoting rehabilitation, and improving the quality of life. WBV exercises relevantly increase the muscle strength, muscle power, and the bone mineral density, as well as improving the postural control, the balance, and the gait. An important number of publications are found in the PubMed database with the keyword “flexibility” and eight of the analyzed papers involving WBV and flexibility reached a level of evidence II. The biggest distance between the third finger of the hand to the floor (DBTFF) of a patient with metabolic syndrome (MS) was found before the first session and was considered to be 100%. The percentages to the other measurements in the different sessions were determined to be related to the 100%. It is possible to see an immediate improvement after each session with a decrease of the %DBTFF. As the presence of MS is associated with poorer physical performance, a simple and safe protocol using WBV exercises promoted an improvement of the flexibility in a patient with MS.

## 1. Introduction

### 1.1. Physical Inactivity and Physical Activity

Physical inactivity is a strong health problem, mainly in developed countries with undesirable consequences to the society due to several facilities. On the other hand, physical activity has important consequences to the health [1–3]. Investigations have shown a strong inverse relationship between physical activity, as habitual exercise, and the risk of coronary disease, cardiac events, and cardiovascular death prevention [1, 4, 5]. Moreover, authors [6, 7] have suggested that exercise may provide some protection against breast, intestinal, prostate, endometrial, and pancreatic cancer.

### 1.2. Physical Activity, the Prevention of Diseases, and the Importance to Patient with Metabolic Syndrome

Considering the patients with osteoporosis, exercise is associated with a decreased risk of hip fractures [8, 9]. Compared to a weight loss diet alone, diet related exercise or exercise and resistance training brings a relevant and important reduction in body fat and enhanced preservation of body lean mass [10]. Metabolic syndrome is defined by an interconnected physiological, biochemical, clinical, and metabolic factors that directly increase cardiovascular risks, such as alterations in the level of the lipids in the plasma, arterial hypertension, central adiposity, and insulin resistance and hyperglycemia [11, 12]. It is well know that regular exercise is a nonpharmacological therapeutic intervention with an enormous range of benefits, including reduced morbidity and mortality of atherosclerotic disease, heart failure, type 2 diabetes, and chronic obstructive pulmonary disease, as well as many other age-related chronic disorders [11, 13, 14]. In addition, the changes in lifestyle and especially in the level of physical activity may help in the treatment and prevention of metabolic syndrome [15]. Other authors have pointed out that aerobic exercise may improve glycemic control and insulin sensitivity and may prevent the development of type 2 diabetes in high-risk groups [16, 17].

Physical fitness components related to health, such as muscle strength and mass, play a significant role in carrying out motor tasks, reducing the risk of falls, and having repercussions in health, longevity, and quality of life of elderly people [18, 19]. Consequently, some studies showed a possible association between muscle strength and decreasing in cardiovascular risk factors, metabolic syndrome, high blood pressure, obesity, and early death [18–22].

It is important to consider the investigation reported by Beavers et al. [23] who have suggested that the presence of metabolic syndrome is significantly associated with poorer physical performance in older adults. In addition, Leite Vieira et al. [24] have reported in an investigation that elderly women with the metabolic syndrome have higher metabolic risk profile and lower functional capacity, muscle strength, lower limb power, and flexibility as compared to women without the metabolic syndrome.

### 1.3. Physical Activities and the Risk of Injuries

Although a wide range of physical activities is available to patients with diseases and the exercises, only a limited number of scientific information had shown the superiority of a kind of activity that would lead to relevant health benefits [25, 26]. Moreover, the undesirable risks related to some types of exercises [27] determine investigations about new possibilities of exercises that could bring benefits to the individual without risks or with minimal possibilities of injuries.

### 1.4. Whole Body Vibration Exercise and Biomechanical Parameters

The exercises in the whole body due to the exposition to energy as vibrations (whole body vibrations exercise—WBV) generated in oscillating/vibratory platform that is transferred to a subject that is in direct contact with the platform seem to bring various benefits [28–30].

There are various devices of platforms that can be used to transfer energy when the individual, in general, is with the feet on the teeterboard of the platform. However, two of them are widely used, as (i) the teeterboard of the platform goes up and down synchronously and (ii) the right side of the teeterboard goes up when the left side goes down in a side-alternating way (and vice versa) [28, 30].

Vibrations, defined as an oscillatory motion, can be generated in oscillating platforms and transmitted, in general, by the feet to whole body of a person [28, 30]. Biomechanical parameters, as frequency and amplitude of the sinusoidal vibration, can be manipulated by the professional that is supervising the clinical procedure. The duration of the work, as well as the time to rest, the number of sets in a session, and the number of sessions, is also controlled. All these conditions depend on, mainly, the clinical and physical conditions of the patient [31]. In sport, Issurin [32] has reported that mechanical vibration can be used as a massage tool and/or for training purposes. This author discusses that two varieties of vibration training can be distinguished: strength exercises with superimposed vibratory stimulation and motor tasks performed under whole body vibration (the WBV training).

### 1.5. Whole Body Vibration Exercise, Potential Biological Effects, and Improvement of Various Clinical Disorders

Authors have demonstrated that WBV exercises might improve muscle strength [33], bone mineral density [34, 35], postural control [33], and muscle power [36]. Moreover, the health-related quality of life is increased and the fall risk is decreased [37]. Improvement of gait and balance with WBV has been shown in a population of nursing home residents [37]. The exercises produced by these vibrations in the human body have also been used successfully to treat patients with some diseases related to the impairments involving the central nervous system, as cerebral palsy [38], multiple sclerosis [39], spinal cord injury [40], and stroke [41]. Fuermaier et al. [42] have published a very important finding that may be of interest to the patient with attention deficit hyperactivity disorder (ADHD). These authors have demonstrated that WBVE improves cognitive performance of healthy individuals as well as of individuals with ADHD. They suggest that the WBV treatment is relatively inexpensive and easy to apply and might therefore be of potential relevance for clinical use. Regterschot et al. [43] have investigated acute effects of passive WBV on executive functions in healthy young adults. Participants underwent passive WBV sessions and nonvibration control sessions while sitting on a chair mounted on a vibrating platform. A passive WBV session was alternated with a control session. After each session, performance on the Stroop color-block test, Stroop color-word interference test, Stroop difference score, and digit span backward task was measured. It is demonstrated that passive WBV has positive acute effects on attention and inhibition in young adults, notwithstanding their high cognitive functioning which could have hampered improvement.

In addition, Gómez-Cabello et al. [44], Di Giminiani et al. [45], Issurin [32], and Issurin et al. [46] have reported an improvement of the flexibility of subjects that have performed WBV exercises.

### 1.6. Whole Body Vibration Exercise and the Improvement of the Flexibility

Flexibility is related to the ability to move joints through their full range of motion (ROM), from a flexed to an extended position and this physical characteristic is highly desired and relevant to a subject to do their daily activities. The flexibility of a joint depends on conditions related to the muscles, ligaments, bones, and cartilage which form the joint. Although the flexibility of a joint can be genetic, it can also be improved by stretching and appropriated exercises [47]. In addition, it is suggested by Rittweger [30] that the stretching could reduce the stiffness and hysteresis of the (i) tendon [48], (ii) alter properties of the intramuscular connective tissue [49], and (iii) possibly alter those of other passive skeletal structures that together define the ROM for a specific joint [50].

Cardinale and Bosco [51] have suggested that the muscle activation due to the WBV may induce improvements in strength and power performance similar to those observed with strength training. As the WBV exercise involves mechanical stretching [52, 53], this fact could justify the increase of the flexibility by the exercise generated by vibration produced in oscillating/vibratory platform and the improvements observed in subjects that have performed WBV [52, 54, 55]. Moreover, an improvement of 8.2% in the sit and reach test has been reported after acute WBV exercise [56]. Similarly, vibration-assisted stretching enhanced the forward split in competitive female gymnasts [57], suggesting improved flexibility in these elite athletes. Di Giminiani et al. [45] have reported that individualized WBV without superimposing other exercises is an effective method of acutely increasing lower back and hamstring flexibility.

Putting together all the information about the potential effects of the WBV and the limitations of the patient with syndrome metabolic, it is important to consider studies involving this kind of exercise and syndrome metabolic patient. The aim of this investigation is to present a short review, using information of the PubMed database, about the findings related to the flexibility in subjects that have performed WBV exercises, and to present a case report of a patient with metabolic syndrome that has improved her trunk flexibility due to a protocol of WBV exercises using lower frequencies.

## 2. Material and Methods

### 2.1. Criteria Used to Find Publications Related to the Utilization of Whole Body Vibration Exercises and Investigations Involving Flexibility

#### 2.1.1. Database Used in This Study

PubMed database was searched on August 7, 2014. PubMed comprises more than 24 million citations for biomedical literature from MEDLINE, life science journals, and online books. Citations may include links to full-text content from PubMed Central and publisher web sites (http://www.ncbi.nlm.nih.gov/pubmed).

#### 2.1.2. Search Strategy Used to Find the Publications Involving WBV and MS

*Searches* were performed using the following keywords: (i) flexibility, (ii) “whole body vibration,” (iii) flexibility and “whole body vibration,” (iv) “whole body vibration” and diabetes, (vi) “whole body vibration” and hypertension, (vii) “whole body vibration” and heart, (ix) “whole body vibration” and metabolic syndrome, (x) flexibility and “whole body vibration exercises,” (xi) flexibility and “oscillating platform,” and (xii) flexibility and “vibratory platform.”

#### 2.1.3. Inclusion and Exclusion Criteria to Select the Publications

Papers were included for analysis if they described a study using whole body vibration generated by an oscillating or vibratory platform in the flexibility of subjects, independently on the clinical conditions. Moreover, the papers must be available in English and reviews were excluded. Articles published before the year 2000 were excluded. Studies involving occupational findings were not also considered. Investigations using medications and whole body vibration were deleted. The publication in which the flexibility was used as a modality of exercise was also deleted. These searches were supplemented with material identified in the references and in the authors' personal files. Data were independently abstracted by four of the authors and disagreements were resolved by consensus.

#### 2.1.4. Level of Evidence of the Selected Papers

The determination of the level of evidence of the selected papers has followed the publication of the “NHMRC additional levels of evidence and grades for recommendations for developers of guidelines” [58].

### 2.2. Case Report and the Protocol Used to Verify the Effect of the Whole Body Vibration Exercises in the Trunk Flexibility of a Patient

#### 2.2.1. Characteristics of the Patient

A 50-year-old patient Caucasian female is an outpatient of the* Hospital Universitário Pedro Ernesto*,* Universidade do Estado do Rio de Janeiro*, Brazil. She has declared to be neither tabagist nor elitist. The clinical examinations (involving physical and laboratory determination) have suggested the diagnosis of metabolic syndrome (MS). This diagnosis of the patient was established for a clinical physician of this multiprofessional team involved in the project who has followed, as inclusion criteria for metabolic syndrome, the guidelines described by the International Diabetes Federation [12]. The patient was not doing another modality of exercise; she was under medication and has followed the recommendations of this clinical physician. As some authors [59, 60] have suggested that the WBV exercises might interfere in some clinical conditions of the patient with MS, participating in an investigation involving the effect of vibrations generated in an oscillating platform was suggested to her.

#### 2.2.2. Ethic Committee and “Written Informed Consent”

This investigation was approved by Ethic Committed under the reference CEP/HUPE 2874/2011, CAAE 0025.0.228.000-11, and the authors have followed the concepts of the Declaration of Helsinki and this research has protected the life, health, privacy, and dignity of the human. All the patients, as well as this patient of this work, read and signed a written informed consent.

#### 2.2.3. Characteristics of the Platform

The oscillating platform (Novaplate fitness evolution, DAF* Produtos Hospitalares Ltda, São Paulo*) used in the study is based in a reciprocating vertical displacements system. It is a side-alternating vibration device working as a teeterboard (28 cm × 58 cm) with amplitude of 0 (zero) in the center of the platform up to the maximum (7.07 mm) in the edge. The displacements are on the left and right side of a fulcrum; while one is up, the other side is down and vice versa. The position of the feet of the subject on the platform will define the amplitude that is used in the exercise and it is controlled during the exercise.

#### 2.2.4. Protocol Used

Sessions of the whole body vibration protocol: in the first session, to aid in the proprioception, the subject was sat in a chair [61] with the feet under the teeterboard of the platform. The frequency used was 5 Hz during 1 min and both feet were in position with amplitude of 2.07 mm in both sides of the teeterboard with a rest of 1 min. This procedure was repeated once again. Then, the patient changed her feet to the amplitude of 4.04 mm in both sides of the teeterboard. The frequency used was the same during 1 min with a rest of 1 min. This procedure was repeated once again. Then, the patient changed her feet to amplitude of 7.07 mm in both sides of the teeterboard, using the 5 Hz of frequency during 1 min, with a rest of 1 min. This procedure was repeated once again.

In the second session, the subject was stood up with both feet under the platform in the amplitude of 2.07 mm of the teeterboard and the frequency used was 5 Hz during 1 min. In this step the man was instructed to be in a comfortable squat position for 1 min of rest. It was repeated once again with a rest of 1 min. Then, the patient still stood up and changing her feet to the amplitude of 4.04 mm sustained the squat comfortable position for 1 min rest. The procedure was repeated once again at the same frequency and amplitude with 1 min of rest. Finally, the patient stood up and changing her feet to the amplitude of 7.07 sustained the squat comfortable position with 1 min of rest. The procedure was repeated once again at the same frequency and amplitude with 1 min of rest.

In the next sessions, the procedures of the second session were repeated in the same conditions in the next sessions; however, in each session, the frequency used was increased in one Hz up to 14 Hz.

In the several steps of the protocol, a physiotherapist was close to the subject.

#### 2.2.5. Measurement of the Anterior Trunk Flexion

Anterior trunk flexion [62] was assessed with the subject in orthostatic position with the knees passively extended followed by carefully of an anterior flexion of the trunk. The distance between the third finger of the hand to the floor (DBTFF) was determined, in each session, just before starting the session and just after the end of each session, as described in the protocol reported in the Section 2.2.4.

The biggest DBTFF was found just before the first session and was considered 100%. The percentages to the other measurements in the different session were determined related to the 100% of the first session.

#### 2.2.6. Determination of Blood Pressure and Heart Rate

An automated device (OMRON, model HEM-7113, China) was used to verify the systolic blood pressure (SBP) and diastolic blood pressure (DBP) (mmHg) and the heart rate (HR) (beats per min-bpm), which were measured on the right arm of seated subjects after a 10-minute rest. These determinations were done just before the first session and just after the last session. Means of three readings of SBP, DBP, and HR were used in the analyses.

#### 2.2.7. Statistical Analysis

Statistical analysis was performed to compare the change in anterior trunk flexibility, SBP and DBP and HR before the first and after the last session of WBV exercise. The level of significance was set at *P* < 0.05.

## 3. Results and Discussion

The findings of this investigation, using information of the PubMed database, reveal that there is interest in evaluating the effect of WBV exercises in the flexibility of subjects. Moreover, a case report of a patient with metabolic syndrome is presented which has significantly (*P* < 0.05) improved her trunk flexibility due to a protocol of WBV exercises using lower frequencies. The SBP, DBP, and HR were not significantly altered (*P* > 0.05). Although other clinical parameters of metabolic syndrome such as blood exam and weight were not determined, these considerations can aid to stimulate the patient with metabolic syndrome to perform WBV exercises. Considering the findings reported by Bogaerts et al. [63] they observed in a community-dwelling elderly that WBV is safe and appears to be efficient to improve the cardiorespiratory fitness and muscle strength. Moreover, Figueroa et al. [64] suggest that WBV may decrease cardiovascular risk in postmenopausal women by improving wave reflection and muscle strength.

Table 1 shows the searches performed in PubMed database. The keyword flexibility rendered more than forty-six thousands publications. The search with the keyword “whole body vibration” rendered more than one thousand publications with general approaches and applications of the WBV. Although no publications were found with “whole body vibration” and “metabolic syndrome,” some publications were found with keywords related to metabolic syndrome, as diabetes and hypertension. Moreover, an important number of articles are observed with the keywords “whole body vibration” and heart. No articles were found in the search in the PubMed with the keywords (a) flexibility and “whole body vibration exercises”, (b) flexibility and “oscillating platform,” and (c) flexibility and “vibratory platform.”

In this study the publications searched with the keywords flexibility and “whole body vibration” that has rendered thirty-two publications were considered. Considering the exclusion criteria, (a) one of these papers was in Russian, (b) three publications before the year 2000, (c) two studies involving occupational findings, (d) four publications involving revisions, (e) one investigation evaluating the effect of medication (alendronate) and whole body vibration [65], (f) two publications [66, 67] in which the flexibility was used as a modality of exercise, (g) two publications, in which the parameter about flexibility was not clearly presented [68, 69], (h) one paper where the effect of the WBV on the flexibility was not clear [70], and (i) one investigation with myoblasts [71] were excluded.

Fifteen publications that reached the inclusion criteria were analyzed considering the effect of the WBV on the flexibility. The frequency and amplitudes used and the mean age and sex of the subjects in the studies and the findings are shown in Table 2.

The level of evidence of the selected papers is also shown in the Table 2.

Sixteen papers involving the flexibility and whole body vibration were analyzed and eight of them were with level of evidence II.

The findings concerning to the systolic blood pressure (SBP) and diastolic blood pressure (DBP) indicated that before the first session, SBP and DBP were 132.15 ± 114.90 mmHg and 74.69 ± 11.03 mmHg, respectively, and after the last session they were 129.35 ± 14.03 and 68.49 ± 16.11 mmHg and no significant difference (*P* > 0.05) was found. The heart rate (HR) before the first session was 74.99 ± 11.01 bpm and after the last session was 73.97 ± 9.94 bpm. No significant difference (*P* > 0.05) was found in the HR. These results indicated that with the protocol used no cardiovascular effect was found in the patient investigated. This fact could be associated with the safety [72, 73] of the WBV exercise and/or the medication that the patient was using.

**Figure 1:**
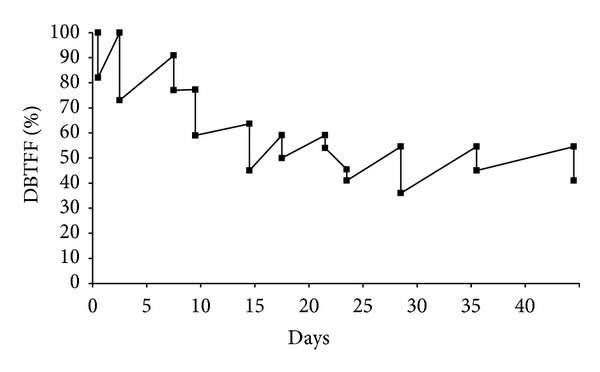
% of the distance between the third finger of the hand of the patient and the floor (DBTFF) in sessions in various days.

Figure 1 shows the values of the %DBTFF in the various sessions. It is possible to see an immediate improvement after each session with a decrease of the %DBTFF. However, it is also shown that the improvement is not maintained. In the next session the %DBTFF increases again. It is highly important to consider that the decrease of the %DBTFF is continuous, as Figure 1 also shows. The difference of the %DBTFF before the first session in comparison with the last session is significant (*P* < 0.05). Moreover, an important reduction is observed up to the 15th day. After this day, the flexibility is maintained up to the end of the protocol. This patient was not evaluated more after the last session.

WBV generated a type of exercise in an oscillating platform that, in appropriated conditions, is safe [72, 73] and has been proposed as clinical intervention in the treatment of several disorders [28–30, 73, 74] as well as to improve the performance of athletes [32, 75–77]. This kind of exercise improves the strength of the muscle [35, 56, 78, 79], bone density [35, 65], cardiovascular parameters [63, 64], body balance [28], flexibility [46, 52, 54, 55, 79–86], and cognition [42, 43] and is a promising treatment method for patients with acute unstable inversion ankle sprains [87]. Some of these improvements might be useful to patients with metabolic syndrome.

Flexibility is regarded as a major component of physical ability and good physical health, particularly the anterior flexibility of the truck [62]. An important number of publications (about fifty thousand) are found in the database PubMed with the keyword “flexibility” (Table 1). Although, there is a relevant number of articles with this keyword, it is necessary to consider that some of them are not related to studies of the flexibility of human beings. Moreover, twelve articles reached the inclusion criteria to be analyzed involving flexibility and whole body vibration and eight of them were with level of evidence II. These papers are discussed in Table 2.

Beavers et al. [23] have suggested that the presence of metabolic syndrome is significantly associated with poorer physical performance in older adults. Considering the risks associated with the physical activity in general [88, 89], the WBV exercises generated by the vibrations obtained in the oscillating platform can be used, under appropriated supervising, without risks in trained and untrained people [30, 32, 90, 91]. Moreover, the clinical findings reveal that WBV exercises might improve muscle strength [29, 90–92], postural control [29, 90], muscle power [30, 36], flexibility [44, 45, 52, 54, 55, 78], and gait and balance [92]. Putting together these findings, the use of this kind of exercises could be useful for patient with metabolic syndrome. The trunk flexibility of a patient with metabolic syndrome has improved after WBV exercises using vibrations generated in oscillating platform with low frequencies (5 up to 14 Hz) (Figure 1). This result is in agreement with other authors that have submitted subjects to similar vibration with high frequencies (15 to 50 Hz) [32, 44, 54].

Although the mechanisms of this effect related to the flexibility are not fully known, previous studies suggest that the improvement in flexibility by WBV is associated with several mechanisms, such as suppression of the central nervous system owing to a decrease in motor neuron pool excitability increasing the blood flow [93–95], decrease in pain sensation [96], and a decrease in musculoskeletal stiffness and inhibition of muscular antagonist [97]. These considerations are relevant and WBV may be favorable for patients with metabolic syndrome.

## 4. Conclusion

An important number of publications (more than forty-six thousand) were found in the database PubMed with the keyword “flexibility.” Eight of the selected papers involving the flexibility and whole body vibration had level of evidence II. This fact could be due to the flexibility be a major component of physical ability and good physical health. WBV is a modality of exercise in an oscillating platform that, in appropriated conditions, is safe and has been proposed as clinical intervention in the treatment of several disorders as well as to improve the performance of subjects, as the improvement of the flexibility. A poor physical performance is observed in the patient with metabolic syndrome and with a simple and safe protocol using WBV exercises, an improvement of the flexibility in a patient with this syndrome was obtained. It is concluded that the WBV exercises could be useful to aid the patient with metabolic syndrome.

## Figures and Tables

**Table 1:** Number of publications studying the use of whole body vibrations in the flexibility of subjects.

Keywords	Number of publications
Flexibility	46,489
“Whole body vibration”	1,158
Flexibility and “whole body vibration”	32
“Whole body vibration” and diabetes	11
“Whole body vibration” and hypertension	3
“Whole body vibration” and heart	60
“Whole body vibration” and “metabolic syndrome"	No items found
Flexibility and “whole body vibration exercises”	Quoted phrase not found
Flexibility and “oscillating platform”	No items found
Flexibility and “vibratory platform”	No items found

**Table 2:** Findings about the effect of the WBV in the flexibility, the frequency used, and the mean age and sex of the subjects in the selected studies.

Publication	Effect in the flexibility/level of evidence (LE)	Age (years)	Sex	Frequency and amplitude
Despina et al., 2014 [84]	Superiority of WBV training, especially in the post 15 measurement, in all flexibility and strength measures, as well as in a number of balance tests in comparison to exercise program performed without vibration. LE II	17.54 ± 0.52	11 women	30 Hz/2 mm

Horstmann et al., 2013 [85]	WBV training may be an alternative or a complementary treatment in patients who do not respond well to eccentric training with improvements in symptoms and pain, structural changes, and muscle flexibility an,d strength of the triceps surae muscle-tendon unit LE II	46.0 ± 6.9	13 men 10 women	13 to 18 Hz/0.4 to 0.6 mm 16 to 21 Hz/0.5 to 0.8 mm

Lee and Chow 2013 [86]	Improvement in lumbopelvic coordination and flexibility after WBV LE III-2	23.2 ± 1.2	10 men	18 Hz/6 mm

Tsuji et al., 2014 [52]	Effect on flexibility was similar with and without vibration stimulus LE II	69.1 ± 2.5	Nine men and 9 women	40 Hz/2–4 mm

Gómez-Cabelloet al., 2013 [44]	WBV group showed better (*P* < 0.005) results in lower-body flexibility and agility compared to the control group LE III-2	75.0 ± 4.7	20 men and 29 women	40 Hz/2 mm

Karatrantou et al., 2013 [54]	Short-term side-to-side WBV training program improved flexibility (*P* < 0.01) LE III-2	20.40 ± 0.27	26 women	25 Hz/6 mm

Wheeler and Jacobson 2013 [55]	No differences (*P* > 0.05) between WBV and light exercise were found for flexibility LE II	20.85 ± 1.81	10 men and 10 women	1 min—20 Hz, 2 min—27.5 Hz, 2 min—35 Hz, 4 min—45 Hz, 1 min—35 Hz

Bunker et al., 2011 [78]	An increase in the flexibility and power output of individual golfers occurs when a WBV warmup bout is performed LE III-3	45 ± 15	10 men	50 Hz/2 mm

Di Giminiani et al., 2010 [45]	Individualized WBV without superimposing other exercises is an effective method of acutely increasing lower back and hamstring flexibility LE II	18.37 up to 24.07	15 men and 19 women	20–55 Hz/1 mm

Feland et al., 2010 [79]	Stretching with vibration on a WBV platform appears to be a good adjunct to static stretching with the potential to enhance retention of flexibility gains LE III-1	23.4 ± 1.7	22 men, 12 women	26 Hz/4 mm

Gerodimos et al., 2010 [80]	Single WBV bout may increase flexibility which persists for at least 15 min and the effects were observed irrespective of frequency and amplitude LE III-3	20.5 ± 1.7	25 women	15–30 Hz/4–8 mm

Jacobs and Burns, 2009 [81]	Short-term WBV standing elicits acute enhancements of lower-extremity muscular torque and flexibility LE III-1	28.6 ± 9.73	10 men and 10 women	Up to 26 Hz

Fagnani et al., 2006 [82]	WBV is a suitable training method to improve knee extension maximal strength, countermovement jump, and flexibility (*P* < 0.001) in female athletes LE II	21 up to 27	26 women	35 Hz/4 mm

van den Tillaar, 2006 [83]	A significant increase in hamstring flexibility was found LE II	21.5 ± 2.0	12 women and 7 men	28 Hz/10 mm

Cochrane and Stannard, 2005 [56]	Acute WBV causes improvement (*P* < 0.05) in the flexibility performance LE II	21.8 ± 5.9	18 women	26 Hz/6 mm

WBV: whole body vibration.

LE: level of evidence.
